# Optic disc pit maculopathy: when and how to treat? A review of the pathogenesis and treatment options

**DOI:** 10.1186/s40942-015-0013-8

**Published:** 2015-08-07

**Authors:** Elad Moisseiev, Joseph Moisseiev, Anat Loewenstein

**Affiliations:** 1 Department of Ophthalmology, Tel Aviv Sourasky Medical Center, 6 Weitzman st., Tel Aviv, 64239 Israel; 2 Affiliated to the Sackler Faculty of Medicine, Tel Aviv University, Tel Aviv, Israel; 3 Department of Ophthalmology and Visual Science, UC Davis Medical Center, Sacramento, CA USA; 4 Department of Ophthalmology, Sheba Medical Center, Ramat Gan, Israel

**Keywords:** Optic disc pit maculopathy, Treatment, Vitrectomy, Review

## Abstract

Optic disc pit (ODP) is a rare congenital anomaly of the optic disc, which can be complicated by a maculopathy associated with progressive visual loss. Optic disc pits are usually unilateral and sporadic in occurrence, and the development of maculopathy is unpredictable with no known triggers. Optic disc pit maculopathy (ODP-M) is characterized by intraretinal and subretinal fluid at the macula, causing visual deterioration. The source of this fluid is still unclear, and several competing theories have suggested it may be vitreous fluid, cerebrospinal fluid, leakage from blood vessels at the base of the pit or leakage from the choroid. The mechanism of pathogenesis of ODP-M has not been fully elucidated, but vitreous liquefaction and traction and pressure gradients within the eye have been implicated to be involved. There are no clear guidelines on the management of patients with ODP-M, and numerous techniques have been described, including laser photocoagulation, intravitreal gas injection, macular buckling and pars plana vitrectomy with many different modifications. The majority of reports describe small series, and as there are no comparative studies there is no consensus regarding the optimal treatment for ODP-M. This review discusses the literature on the possible sources of fluid and mechanisms of pathogenesis in ODP-M, as well as the wide array of treatment modalities and their results. Based on these, a set of recommended key concepts for the timing and choice of treatment for these challenging are presented.

## Introduction

Optic disc pits are considered as part of a spectrum of congenital cavitary anomalies of the optic disc, which also includes optic disc coloboma, morning glory and extrapapillary cavitation [1]. Histologically, an ODP is a herniation of dysplastic retina into a collagen-rich excavation that extends into the subarachnoid space through a defect in the lamina cribrosa [2]. This abnormal structure of the optic disc creates an anomalous communication between the intraocular and extraocular spaces, a feature shared by all congenital cavitary anomalies of the optic disc [1, 3].

ODPs are rare, and occur equally in men and women with an estimated incidence of 1 in 11,000 people [3, 4]. They are typically unilateral, but may be bilateral in up to 15% of patients [4, 5]. The occurrence of ODPs is usually sporadic, but possible autosomal inheritance has been suggested in some pedigrees with multiple affected members [6, 7]. No specific gene has been associated with ODP formation.

ODPs are usually seen as single, oval-shaped depressions at the optic disc. They are most commonly found at the inferotemporal aspect of the optic disc, but may also be found elsewhere, including centrally [4, 8]. Occasionally, an optic disc can have more than one pit [9]. ODPs are usually grayish, but may also be yellow or black [4, 5].

**Fig. 1:**
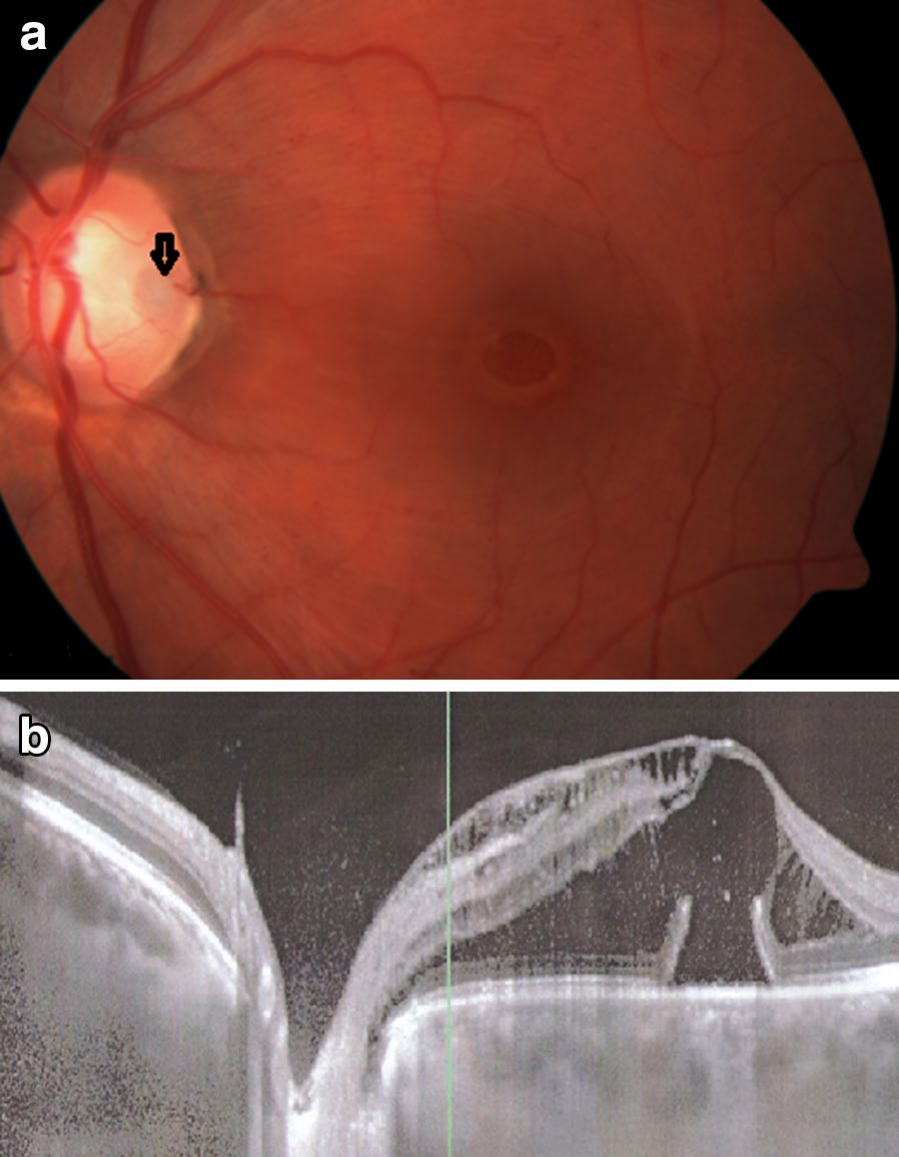
**a** Fundus photograph of the left eye of a 31 year old man with ODP-M. A temporal ODP is noted (*arrow*). VA was 20/200. **b** A horizontal OCT scan through the optic disc and fovea, showing an abundance of intraretinal and subretinal fluid, extending towards the ODP. *ODP* optic disc pit, *ODP-M* optic disc pit maculopathy, *VA* visual acuity and *OCT* optical coherence tomography.

An ODP by itself is usually asymptomatic, and may be found incidentally. Nevertheless, the presence of this defect has been demonstrated to cause visual field defects, most commonly an enlarged blind spot and a paracentral arcuate scotoma [3, 9, 10]. However, when an ODP is complicated by maculopathy, it may cause significant visual deterioration. Optic disc pit maculopathy (ODP-M) is a term used to describe macular changes that occur in the context of an ODP, which include intraretinal and subretinal fluid accumulation, and retinal pigment changes [3, 4]. An example is provided in Fig. 1. Maculopathy occurs in 25–75% of patients with an ODP [3, 9, 11], and usually when the ODP is temporal. There is no known trigger for the development of ODP-M. Since ODP-M usually occurs in the third and fourth decades of life [5, 12], it has been postulated to be associated with posterior vitreous detachment (PVD) [1, 4, 12]. However, ODP-M has also been reported in pediatric patients with no vitreous liquefaction [1, 13].

When ODP-M is present, visual acuity (VA) is usually reduced to 20/70 or worse [4]. Although spontaneous resolution with improvement in vision has been reported [14, 15], the majority of cases have a poor prognosis, with a natural history of gradual worsening and a final VA of 20/200 or worse [16–18]. Therefore, interventions for the treatment of ODP-M are valuable, as they may prevent significant visual loss in these patients. With the advances made in ocular imaging and surgical techniques over the past decades, several theories of ODP-M pathogenesis and interventions for its treatment have been suggested. Despite the improved insight into this unusual pathology, there is currently no consensus regarding its optimal treatment. The purpose of this literature review is to discuss the mechanisms of pathogenesis that may be responsible for ODP-M formation, and the possible treatment options for this challenging entity.

## Review

Although ODP-M is a well-known pathology for several decades, the nature and origin of the fluid that is found intra- and sub-retinally in these patients is still controversial [10, 19]. Several different mechanisms of pathogenesis have been proposed for ODP-M, and these theories have led to a variety of interventions intended to treat it. As ODP-M is a relatively rare diagnosis, most reports include case reports and small series, and there are no large or comparative studies whose results can serve to guide treatment in these cases. The following is a review of the theories regarding the origin of fluid in ODP-M, its pathogenesis and reported treatment options.

### Origin of ODP-M fluid

Over the years, four different possible sources have been proposed for the fluid seen in the intraretinal and subretinal spaces in ODP-M.

The first possible source of fluid is the vitreous. It was first suggested that vitreous fluid can enter the subretinal space through the formation of a macular hole [19, 20]. This initial assumption is most likely erroneous, as macular holes do not typically occur in patients ODP-M [19]. Later works on Collie dogs demonstrated that India ink dye injected into the vitreous was subsequently found in the subretinal space [21, 22]. It should be noted, however, that glycosaminoglycans, which are a component of the vitreous, were not found in the subretinal fluid in Collie dogs [21], and a direct connection between the vitreous and subretinal space through the ODP was not demonstrated by imaging or histology. A histopathologic study on 2 human eyes with ODP-M did report detecting mucopolysaccharides, which are also a component of the vitreous, inside the ODPs [2]. Additionally, several studies reported on the passage of gas or silicone oil from the vitreous cavity to the subretinal space in eyes with cavitary anomalies of the optic disc, including ODP [23–25].

The second possible source of fluid is the cerebrospinal fluid (CSF), which has been proposed to enter the intra- and sub-retinal spaces from the subarachnoid space through the ODP defect. Several optical coherence tomography (OCT) studies have shown direct communication exists between the subarachnoid space and the subretinal space [26–28]. Further evidence supporting this concept is that gas bubbles were reported to percolate out of an optic nerve sheath window after pars plana vitrectomy and gas injection, indicating a continuity between the posterior vitreous cavity and the optic nerve subarachnoid space [29]. Additionally, intracranial migration of silicone oil has been reported in a patient with an ODP who underwent surgery for repair of retinal detachment [30]. A connection between the subarachnoid and subretinal spaces has also been reported in morning glory anomaly [31]. It should be noted, however, that one study using intrathecal fluorescein did not demonstrate a connection between the subarachnoid and subretinal spaces [32].

The third possible source of fluid is leakage from blood vessels at the ODP [33]. This concept was based on the finding of late hyperfluorescence at the ODP in fluorescein angiography, as well as in the area of macular elevation in eyes with ODP-M [34, 35]. However, some patients with ODP-M do not demonstrate this late hyperfluorescence [9].

The fourth possible source of fluid is from the choroid, through the Bruch’s membrane and peripapillary atrophy [36]. This source is unlikely, as subretinal fluid is often not observed in other diseases that cause significant chorioretinal atrophy [9, 19].

### ODP-M mechanism of pathogenesis

Beyond the controversy regarding the source of fluid in ODP-M, the mechanism of pathogenesis for this pathology is also unclear. ODPs are congenital, but the development of ODP-M has no known triggers, and can occur at any age, from early childhood to the ninth decade of age [19]. Several mechanisms of pathogenesis have been proposed, but none has been proven beyond all doubt.

As previously mentioned, ODP-M typically occurs in the third and fourth decades of life [5, 12], which is also the time of onset of progressive vitreous liquefaction. Therefore, it has been proposed that vitreous traction is responsible for ODP-M development. In a relatively large series of patients with ODP, the majority of those with ODP-M had PVD while the majority of those who did not had not yet undergone PVD [9]. Additionally, spontaneous resolution of ODP-M has been reported following completion of PVD [11]. Treatment of ODP-M with pars plana vitrectomy (PPV) including PVD induction is an effective option, suggesting that relieving vitreous traction over the ODP can achieve resolution of ODP-M [1, 37, 38]. Several OCT studies have demonstrated vitreous strands over ODPs [28, 30, 40]. Several reports describe a membrane over the ODP [41–43], and it has been noted that ODP-M can develop after this membrane disappears, presumably due to vitreous traction over it [44, 45]. On the other hand, ODP-M can occur in pediatric patients before the onset of any changes in the vitreous, and also in adults without PVD [1, 13, 19]. There have also been several OCT studies that did not demonstrate any evidence of vitreous traction over OPDs [37–40, 46]. Furthermore, ODP-M can recur following PPV, suggesting that vitreous traction may not be required for its formation [40].

Another theory is that pressure gradients within the eye cause migration of fluid from the vitreous into the subretinal space. A normal eye is a closed system, without significant differences in pressure between various compartments. In an eye with an ODP, a pressure gradient can exist because the intracranial pressure is transmitted to the ODP via the CSF. Thus, when the intracranial pressure is low vitreous fluid is drawn into the ODP, and when it rises the fluid is pushed back into the eye, and can dissect under or within the retina [1, 47]. This mechanism can also explain the intraretinal, subretinal and intracranial migration of vitreous substitutes (gases or silicone oil) in eyes with ODPs [23–25, 29, 30].

Regardless of the source of fluid and exact pathophysiologic mechanism of ODP-M, a sequence of retinal fluid accumulation and progression of its formation has been described by Lincoff et al. [17], which is generally accepted [1, 10, 19]. First, fluid from the ODP creates a schisis-like inner retinal separation, associated with a mild cecocentral scotoma. Then, an outer layer macular hole develops beneath the inner layer, associated with a dense central scotoma. The fluid then dissects subretinally creating an outer retinal detachment. This sequence has been supported by OCT studies [26, 27, 46]. It has also been previously reported that virtually all ODP-M cases have intraretinal fluid in the outer nuclear layer, and claimed that none have isolated subretinal fluid [48], supporting the concept that the fluid first enters the inner retinal layers and only later makes its way to the subretinal space. It has been suggested that as fluid accumulates intraretinally in eyes with ODP-M, a pressure gradient is formed that is directing it into the retina and to the subretinal space [49].

### Treatments for ODP-M

Treatment for ODP-M is warranted as the majority of cases suffer gradual deterioration with significant visual loss [16–18]. A multitude of interventions have been designed for the treatment of ODP-M, but none has been established as the treatment of choice.

Early reports described treating ODP-M with oral corticosteroids. This treatment was not effective, and resorbed fluid tended to recur following discontinuation of the corticosteroids [1, 50]. Therefore, this is no longer considered an appropriate treatment option.

Laser photocoagulation at the temporal disc margin has been proposed as a treatment for ODP-M, with the reasoning that the laser scars will create a chorioretinal adhesion which will act as a barrier between the ODP and the subretinal space [33]. Initially, ODP-M patients were treated with Xenon laser and did not improve [33]. Later patients were treated with Argon laser, and small series reported absorption of fluid and reattachment of the retina in some patients [12, 51]. The time for improvement was variable and often long [50, 51], and the location of the laser treatment could also cause significant visual field defects.

Intravitreal gas injection has been proposed as a treatment option for ODP-M, with the reasoning that pneumatic displacement will cause reattachment of the macula and improve VA [52]. This technique was used in small series, and resulted in visual improvement, although retinal reattachment was only achieved in about half of the cases [52, 53]. One small series of patients treated with a combination of intravitreal gas injection and laser photocoagulation temporal to the disc reported visual improvement and reduction in fluid in all eyes, and complete resolution of intraretinal and subretinal fluids in 75% of eyes [54]. These studies are summarized in Table 1.

**Table 1:** A summary of the series describing treatment of ODP-M with intravitreal gas injection

References	No. of cases	Technique	Main results	Complications
Lincoff et al. [52]	3	C2F6 gas injection (no laser)	Initial improvement but later recurrence	None
Akiyama et al. [53]	8	SF6 gas injection (no laser)	50% resolution of intra/sub-retinal fluids More than 1 injection often necessary	None
Lei et al. [54]	8	C3F8 gas tamponade with laser photocoagulation	75% resolution of intra/sub-retinal fluids VA improved in 7 eyes	None

*ODP-M* optic disc pit maculopathy, *VA* visual acuity, *C2F6* perfluoroethane, *SF6* sulfur hexafluoride and *C3F8* perfluoropropane.

An alternative approach proposed for the treatment of ODP-M is macular buckling surgery. This procedure includes an implant that is fixed to the posterior aspect of the globe along the 6-to-12 o’clock meridian, creating a buckling effect under the macula [55]. This technique has been reported to achieve complete resolution of fluid in about 85% of cases, as well as significant improvements in VA and visual fields [45, 55–57]. Long-term follow up studies of patients treated with this technique have demonstrated that success was maintained for over 10 years, with very low rates of complications or recurrences, and long-term visual improvement [58]. Additionally, restoration of foveal outer retinal layer structure was documented by OCT [59]. These results are impressive, but it should be noted that the surgical technique is complicated (intraoperative B-scan is required for exact positioning of the macular buckle). The technique has not gained popularity since its introduction 20 years ago, and all reports on its results are from the same group.

The predominant approach for the treatment of ODP-M is pars plana vitrectomy (PPV). The majority of the published literature on ODP-M is focused on PPV techniques, which have evolved along with the advances made in surgical technology. Several anecdotal reports from the late 1980’s and early 1990’s described successful anatomical and visual restoration in patients with ODP-M who underwent PPV with or without endolaser to the temporal disc margin and gas tamponade [60–63]. In a series of 10 patients treated with PPV, laser and gas tamponade, visual improvement was achieved in 90% and complete resolution of fluid in 70% [64]. These early reports of successful treatment of ODP-M with PPV paved the way for numerous attempts to improve this technique.

It has been proposed that induction of complete PVD during surgery is essential for relief of traction required to achieve macular reattachment [65]. This was initially reported anecdotally [65, 66], and later supported by two different series of 11 patients [37, 67]. García-Arumí et al. [67] reported on 11 ODP-M patients treated with PPV, induction of PVD, laser and gas tamponade, achieving anatomical resolution and significant visual improvement in all cases, with only 2 recurrences. Hirakata et al. [37] reported on 11 ODP-M patients treated with PPV, induction of PVD and gas tamponade without laser, achieving complete resolution of fluids in 10 of them. Based on these results, it has been suggested that laser photocoagulation at the temporal disc border may not be required for successful treatment of ODP-M. Gas tamponade has been performed in the vast majority of published cases, as it is used to create a temporary barrier blocking the passage of fluid thorough the ODP. Interestingly, anecdotal cases have been reported of surgical interventions without gas tamponade that failed to improve ODP-M, but when intravitreal gas was injected in a second procedure the fluids have resolved [68, 69]. In another series of 7 patients treated with PPV and PVD induction without laser or gas tamponade, VA had improved but the fluids did not resolve completely [38]. This evidence further strengthens the importance of gas tamponade in the treatment of ODP-M. Silicone oil has been very infrequently used in patients with ODP-M, but when used it had been effective [70]. It had also been reported to be effective in a persistent case that failed previous surgical treatment [71]. However, it should be noted that silicone has also been reported to migrate intracranially through an ODP [30].

One of the early reports on PPV for ODP-M described internal submacular fluid drainage [63]. Although successful treatment has been reported in the absence of this surgical maneuver [37, 61, 62, 64–68], and even when it has been attempted unsuccessfully [72], it has been used by several surgeons. A few cases of PPV with induction of PVD, endolaser, submacular drainage and gas tamponade have been reported to achieve complete resolution of fluid which was maintained over 2 years since surgery [73]. Additionally, a technique of subretinal drainage using a 42-gauge needle without the need for retinotomy has been described, and used successfully in one patient [74]. Recently, intraoperative OCT technology has been shown to be capable of assisting the surgeon in performing effective subretinal drainage [75].

Another controversial aspect is the necessity to peel the internal limiting membrane (ILM) in patients with ODP-M. It has been suggested to be in important component of the surgical treatment of ODP-M [76], and a few cases of PPV with PVD induction, ILM peeling and tamponade with gas or air have been reported to achieve successful resolution of ODP-M [77, 78]. One case in which PPV with PVD induction, laser and gas tamponade had failed was reported to have resolved following a second intervention in which the ILM was peeled [79]. Rizzo et al. [80] reported on ten patients with ODP-M who were treated by PPV, induction of PVD, ILM peeling, laser and gas tamponade, with visual improvement in 90% of them and complete resolution of fluids in 50%. Shukla et al. [81] reported on seven patients treated similarly, with complete resolution achieved in six of them, and five achieving final VA of 20/30 or better. It has been suggested that patients whose ODP-M consists of a multi-layer schisis are more difficult to treat than those whose maculopathy consists predominantly of subretinal fluid, and that in such cases ILM peeling should be performed to achieve optimal outcomes [82]. On the other hand, good results have been reported without ILM peeling and it may not be required for successful treatment of ODP-M [1, 37–39, 64, 67, 83, 84].

Some authors have proposed that in addition to inducing PVD, the surgeon should look for any glial tissue overlying the ODP and carefully peel it off during surgery. This maneuver has been described infrequently, and the ODP-M had resolved [85]. In one report on 9 eyes with ODP-M treated by PPV, induction of PVD, laser and gas tamponade, complete resolution was achieved in 6 of 6 eyes in which glial tissue was removed from the ODP, compared to only 2 of 3 eyes in which this was not done [39]. Additionally, there are several reports that suggested sealing of the ODP during surgery to prevent passage of fluid into the intraretinal and subretinal spaces. The first description was of a case successfully treated with injection of autologous platelets over the ODP [86]. Other techniques designed to seal the ODP include using an autologous scleral flap [87], inverting peeled ILM into the ODP [88], and using Tisseel fibrin sealant [89].

A recent series by Ooto et al. [90] reported 18 eyes with ODP-M treated with PPV and a creation of inner retinal fenestrations (which are partial thickness retinotomies) just temporal to the ODP. Cortical vitreous was removed in only five eyes, fluid-air exchange was performed in one eye, and ILM peeling or laser were not performed in any eye. This technique is based on the theory that there is a pressure gradient pushing fluid from the inner retina into the submacular space, and that through these fenestrations it will be diverted back into the vitreous [49, 90]. This is the largest series of ODP-M eyes treated with a single technique, and its results are very good—complete resolution of foveal fluid was achieved in 17 (94%) eyes, and VA was significantly improved with 10 (56%) eyes achieving 20/30 or better [90]. One previous case report described resolution of ODP-M following a partial thickness retinotomy that connected an intraretinal schisis cavity to the vitreous space [91]. It should also be noted that a case of premature closure of such a fenestration and persistence of ODP-M has been reported [92]. Future studies of this technique are needed to support its efficacy.

A summary of the major published series of PPV treatments for ODP-M is provided in Table 2.

**Table 2:** A summary of the major published series of PPV treatments for ODP-M

References	No. of cases	PPV gauge	Surgical technique	Main results	Complications
Taiel-Sartral et al. [64]	10	20	PPV+laser+gas	VA improved in 90% Mean VA improvement of 6.7 lines Complete resolution in 70% 1 recurrence (10%)	None
García-Arumí et al. [67]	11	20	PPV+PVD+laser +gas	82% gained ≥2 lines of VA Mean final VA 20/32 Complete resolution in all cases 2 recurrences (18%)	None
Hirakata et al. [37]	11	20	PPV+PVD+gas	VA improved in 64% Complete resolution 91% Showed that laser may not be required for surgical success	1 patient developed a dense inferotemporal scotoma 1 patient developed an area of retinal atrophy 1 patient had an intraoperative retinal break, treated with laser
Ghosh et al. [71]	7	20	PPV+PVD+laser +gas	4 (57%) gained ≥2 lines of VA 4 (57%) required second procedure Silicone oil was successful in a refractive case	1 patient developed postoperative glaucoma and cataract
Rizzo et al. [80]	10	23/25	PPV+PVD+ILMP +laser+gas	70% gained ≥2 lines of VA Complete resolution in 50% First report with small gauge PPV	1 complication—macular hole
Hirakata et al. [38]	8	20/25	PPV+PVD (no gas)	VA improved in 7 (88%) eyes Complete resolution in 88% Suggested that gas tamponade may be less important than induction of PVD	None
Shukla et al. [81]	7	23	PPV+PVD+ILMP +laser+gas	6 (86%) eyes gained ≥2 lines of VA Final VA ≥20/30 in 5 (71%) eyes	4 eyes had full thickness macular holes at 1 month, but it closed in 3 of them 2 patients had transient elevated intraocular pressure 1 patient developed cataract
Avci et al. [83]	13	23	PPV+PVD+laser +gas	11 (85%) eyes gained ≥2 lines of VA Final VA ≥20/40 in 6 (46%) eyes Complete resolution in 92% Suggests ILMP may not be required for surgical success	2 patients developed cataracts
Gregory-Roberts et al. [39]	9	N/A	PPV+PVD+glial tissue removal+laser +gas	Complete resolution in 8 (89%) eyes Higher rate of complete resolution when glial tissue was removed over the pit	1 patient developed cataract
Ooto et al. [90]	18	25	PPV+intraretinal fenestration	Complete resolution in 94% VA improved in all eyes 11 (61%) eyes gained ≥3 lines of VA Final VA ≥20/30 in 10 (56%) eyes	None

Presented are the only studies that included more than five patients with ODP-M that were treated using a uniform PPV technique. *PPV* pars plana vitrectomy, *ODP-M* optic disc pit maculopathy, *PVD* induction of posterior vitreous detachment, *laser* laser photocoagulation temporal to disc, *gas* gas tamponade, *ILMP* internal limiting membrane peeling and *VA* visual acuity.

## Conclusions

Although ODPs are rare, it is likely to assume that every vitreoretinal surgeon will encounter several patients with ODP-M during his or her career. These cases are challenging to manage. Compared to other indications for vitreoretinal surgery, there is a paucity of literature on this unique pathology, the majority of which includes case reports and small series with no comparative studies. This makes patient counseling, expectation setting and decision making regarding the timing and choice of surgical intervention very difficult. There are no established guidelines for the treatment of ODP-M, and no consensus on the mechanism of pathogenesis or the optimal surgical technique. Additionally, many of the proposed surgical techniques are challenging to perform. Based on the literature review, we suggest the following key concepts for the timing and choice of treatment of patients with ODP-M.

1. Patients with ODPs that are diagnosed incidentally should be counseled on the risk for development of ODP-M. Although rates vary from 25 to 75% [3, 9, 11] and there are no known triggers for it, patients should be encouraged to return for examination in any case of visual deterioration in an affected eye. Routine yearly follow-up may also be advised. There are no prophylactic treatments, and prophylactic peripapillary laser is not recommended as it has not been ever studied as a preventative measure, and may be associated with creation of significant visual field defects in asymptomatic eyes [1].
2. Although spontaneous resolution is possible in as many as 25% of cases and visual improvement is possible [14, 15, 93], observation will generally lead to significant visual loss [16–18]. Initially it has been recommended to wait for spontaneous resolution for up to 3 months before considering surgery [4, 33], but today currently observation is deemed unjustified [10, 80]. This is especially true in the presence of progression of fluid accumulation or visual loss.
3. The most commonly used procedure for the treatment of ODP-M is PPV, especially in recent years. Treatment with laser alone was not very successful [8, 50, 51], and should currently be reserved for patients who cannot undergo any surgery due to their systemic condition. Although successful management has been reported with intravitreal gas injections and macular buckling, limited experience exists for these procedures [45, 52–59]. Macular buckling for ODP-M is a very technically challenging procedure, but experienced surgeons can consider it in such cases.
4. When PPV is performed, it is worthwhile to induce complete PVD to relieve any traction on the ODP [65–67]. Triamcinolone acetate may be used to ascertain that all vitreous has been removed from the posterior pole. Gas tamponade is also important, and acts as a barrier that blocks passage of fluid through the OPD, and has been used in the majority of cases in published literature. Perfluoropropane (C3F8) and sulfur hexafluoride (SF6) gases are likely to be equally effective in these cases [84]. We note that both induction of PVD and gas tamponade are surgical techniques that vitreoretinal surgeons are very experienced in as they are commonly performed in surgery for other indications. These two elements are probably the most important in achieving success with PPV for ODP-M. Although the source of fluid and exact pathogenesis of ODP-M are unknown, these steps provide a “theoretical broad coverage” as they will relieve vitreous traction and create a barrier for pressure gradients and the passage of fluid.
5. Additional elements of surgery, such as peripapillary laser, ILM peeling, subretinal drainage, peeling of glial tissue and sealing of the ODP are controversial. Both success and failure of surgical management have been reported with and without them, and available data is not enough to conclude that any of them are associated with better outcomes. Therefore, these should be performed at the discretion of the surgeon, following a discussion of their potential advantages and risks with the patient.
6. Limited vitrectomy with intraretinal fenestration is a recently described technique that has promising results [90]. Future studies are required to support its efficacy and compare it to PPV with induction of PVD, gas tamponade and any of the additional elements discussed. It is possible that use of intraoperative OCT can facilitate the creation of the fenestrations. We expect such studies to be published in coming years.
7. The most important issue to discuss with ODP-M patients in whom surgery has been recommended is that the visual recovery is a slow and long process. All surgical techniques have been reported to achieve complete resolution of fluid and significant visual improvement, but these were documented after at least 3 months and generally only after 6–12 months from surgery [37, 38, 41, 45, 55–57, 64, 67, 70–73, 80–84, 90]. This is very important for appropriate setting of patient expectations prior to surgery.
8. The majority of reports describe successful outcomes that have been maintained long term, with relatively low rates of ODP-M recurrence and very few postoperative complications.
